# Age of Onset of RNA Toxicity Influences Phenotypic Severity: Evidence from an Inducible Mouse Model of Myotonic Dystrophy (DM1)

**DOI:** 10.1371/journal.pone.0072907

**Published:** 2013-09-05

**Authors:** Jordan T. Gladman, Mahua Mandal, Varadamurthy Srinivasan, Mani S. Mahadevan

**Affiliations:** 1 Department of Pathology, University of Virginia, Charlottesville, Virginia, United States of America; 2 Parexel International, Bethesda, Maryland, United States of America; Colorado State University, United States of America

## Abstract

Myotonic dystrophy type 1 (DM1) is the most common muscular dystrophy in adults. It is caused by an expanded (CTG)n tract in the 3′ UTR of the *Dystrophia Myotonica Protein Kinase* (*DMPK*) gene. This causes nuclear retention of the mutant mRNA into ribonuclear foci and sequestration of interacting RNA-binding proteins (such as muscleblind-like 1 (MBNL1)). More severe congenital and childhood-onset forms of the disease exist but are less understood than the adult disease, due in part to the lack of adequate animal models. To address this, we utilized transgenic mice over-expressing the *DMPK* 3′ UTR as part of an inducible RNA transcript to model early-onset myotonic dystrophy. In mice in which transgene expression was induced during embryogenesis, we found that by two weeks after birth, mice reproduced cardinal features of myotonic dystrophy, including myotonia, cardiac conduction abnormalities, muscle weakness, histopathology and mRNA splicing defects. Notably, these defects were more severe than in adult mice induced for an equivalent period of exposure to RNA toxicity. Additionally, the utility of the model was tested by over-expressing MBNL1, a key therapeutic strategy being actively pursued for treating the disease phenotypes associated with DM1. Significantly, increased MBNL1 in skeletal muscle partially corrected myotonia and splicing defects present in these mice, demonstrating the responsiveness of the model to relevant therapeutic interventions. Furthermore, these results also represent the first murine model for early-onset DM1 and provide a tool to investigate the effects of RNA toxicity at various stages of development.

## Introduction

Myotonic dystrophy type 1 (DM1) occurs in 1∶8000 adults making it the most common autosomal dominant muscular dystrophy. It is caused by a (CTG) triplet repeat expansion in the *DMPK* gene resulting in nuclear localization of mutant mRNA. This mRNA then forms ribonuclear foci (RNA foci), which are thought to be deleterious due to their interaction with, and sequestration of, RNA-binding proteins such as MBNL1. Additionally other proteins such as CUGBP1, NKX2-5, Staufen1, and SHARP have been shown to be improperly expressed in tissues from people with DM1[1]–[4]. As a result of this “toxic” RNA and the resulting disruption in the normal molecular make-up, symptoms including myotonia, muscle wasting, weakness and histopathology, cardiac conduction defects, cataracts, and insulin resistance occur in individuals with DM1 [5]–[7]. More severe congenital and childhood-onset forms of myotonic dystrophy also exist with many of the same symptoms found in adult onset DM1; however they also present with additional complications.

Congenital DM1 is considered the most severe form of the disease. The inheritance is almost always maternal in origin and is associated with extreme expansion of the (CTG)n tract [8]. The condition is evident at birth and can even be detected in prenatal ultrasound as a lack of fetal movements. When born, the infants are hypotonic, have poor muscle development, suffer from respiratory difficulties, can have craniofacial abnormalities, and often have severe mental retardation [9]–[12]. Mortality is high in this group and extensive medical intervention is required. There is however a milder form of myotonic dystrophy that occurs in childhood. While still being more severe than the adult onset form, it represents a distinct clinical group within the spectrum of myotonic dystrophy [13]–[15]. Unlike congenital myotonic dystrophy, individuals with childhood-onset myotonic dystrophy do not have prenatal abnormalities, nor delayed early motor development and, if present, only have mild hypotonia or respiratory problems [9]–[12], [14]. Childhood-onset DM1 patients usually have myotonia and frequently have mental handicaps such as a decrease in mean IQ and a range of psychosocial difficulties [13], [15]. As patients age they tend to develop features seen in adult onset DM1.

The Mahadevan laboratory previously generated a DM5 mouse model of RNA toxicity in which transgenic animals over-express the green fluorescent protein (GFP) mRNA fused to the *DMPK* 3′UTR-(CTG)_5_ under the control of a doxycycline-inducible *DMPK* promoter [2], [16]. Although not a mouse model with an expanded (CTG) repeat transgene, the DM5 model mimics nearly all features of human disease after induction of transgene expression. This includes cardiac conduction deficits, myotonia, muscle histopathology, muscle weakness, increased CUGBP1 levels, and mRNA splicing defects [2], [16]. This animal model led to the discovery that when expression of the toxic RNA is silenced, disease symptoms such as myotonia, cardiac conduction defects, and mRNA splicing abnormalities can be corrected [16]. In addition we have found that the rate of disease progression can be modified by altering the copy number of the DM5 transgene and the concentration of the administered doxycycline. In this study we have developed a mouse model of early-induction DM1 (EDM1) with clinical features that resemble the human condition. In these mice, the early age of disease onset leads to a markedly more severe disease phenotype when compared to mice in which RNA toxicity is induced after they reach adulthood. Additionally we found improvement in skeletal muscle myotonia and splicing defects after over-expression of MBNL1, revealing the possible role of MBNL1 deficiency in the pathology of these mice and also demonstrating the potential of this mouse model for testing compounds aimed at treating the disease phenotypes associated with DM1.

## Materials and Methods

### Mouse Lines

All procedures involving experimental animals were performed in compliance with the University of Virginia and local animal welfare laws, guidelines and policies. This study was carried out in strict accordance with the recommendations in the Guide for the Care and Use of Laboratory Animals of the National Institutes of Health. The protocol was approved by the University of Virginia Institutional Animal Care and Use Committee (Protocol Number: 3218). Transgenes and phenotypic characterization of transgenic mice are described elsewhere [16]. The DM5 line (DM5-313) expresses the GFP-*DMPK* 3′UTR transgene under the control of a tetracycline responsive human *DMPK* promoter. The mice are normal prior to transgene induction. Transgenic mice were induced with 0.2% doxycycline in drinking water. To generate EDM1 animals, adult DM5^+/−^ mice (2 months old) were exposed to doxycycline to induce the DM1 phenotype for 2 months before breeding. The females, both wildtype and DM5 were then exposed to doxycycline during breeding, pregnancy and weaning. The resultant EDM1 pups continued to be exposed to doxycycline until the end of the experiment.

For analyses of transgene expression during embryonic development, DM5^+/+^ males were bred to wildtype FVB females which were administered 0.2% doxycycline in their drinking water, and embryos were collected at 6, 10, 12, and 20 days of gestation and used for RNA analyses by qRT-PCR. Wildtype mouse embryos were also collected at 6–10 days of intrauterine life to study the onset of *Dmpk* expression using whole mount *in situ* hybridization. PCR products containing the *Dmpk* gene were used to generate antisense and sense probes and labeled using the Digoxigenin RNA Labeling kit from Roche Applied Sciences. Probe generation, purification and modified in situ hybridization procedures were done as previously described [17].

### Mass, Grip Strength, ECG and EMG

The average weights of the mice used in the experiments is listed in Table S1in File S1. For grip strength analysis, the mouse is trained to grip a bar attached to a force transducer, and steady pressure is applied as the mouse is pulled until it releases the bar. The maximum force of the grip is assessed by averaging six values obtained for each mouse. Differences in grip strength due to differences in mouse weight are corrected by normalizing the average grams of force by the body mass for each mouse. For EMGs and ECGs, mice were anaesthetized with intraperitoneal midazolam (1.25 mg/kg) and ketamine (100 mg/kg) and kept under a warming lamp and warming blanket during the entire procedure. Myotonia was measured by EMGs performed on the hamstring, soleus, and gastrocnemius muscles as described previously with the results from the gastrocnemius used for scoring [16]. Three lead ECGs were performed using a BioAmp/Powerlab from ADInstruments [16].

### Tissue Collection and Histology

Mice were euthanized and tissues were flash frozen in liquid nitrogen chilled isopentane. Serial cryostat sections (6 um) of skeletal muscles were prepared unfixed and stained with hematoxylin and eosin (H&E) and morphology was assessed using an Olympus IX 50 inverted microscope. Images were captured with a CCD camera and presented using Photoshop 5.5. AxioVision 4.8.2.0 (Carl Zeist MicroImaging) was used to assess tissue histology and determine cross-sectional fiber diameters.

### Protein Blotting

Total protein extracts from frozen tissues were made using standard protocols in RIPA buffer (50 mM Tris-HCl, pH 7.4, 150 mM NaCl, 1% NP40, 0.5% Na-deoxycholate, 0.1% SDS) and protease inhibitor (Roche). Blots were blocked in 5% Milk/TBST. Antibodies for GAPDH (Ambion, Inc.), CUGBP1 and MBNL1 (Abnova, Taiwan) were used for western blotting. Blots were scanned and relative protein expression was quantified using Image Quant5.1.

### RNA-Immunoprecipitation (RNA-IP)

Mouse skeletal muscles were used for RNA-IP with a MBNL1 monoclonal antibody (Abnova, Taiwan) without cross-linking agents. Tissue was snap frozen, ground in a mortar and pestle and resuspended in Sanford IP buffer (10 mM HEPES pH 7.3, 100 mM NaCl, 3 mM MgCl2, 0.5% NP-40, 0.5% Triton X-100) supplemented with, RNase inhibitor to a final concentration of 100 units/mL, protease inhibitor to a final concentration of 1×, phosphatase inhibitor to a final concentration of 1×, and DTT to a final concentration of 1 mM. The lysate was then immunoprecipitated either with anti-MBNL1 or isotype control using magnetic beads (GE Healthcare, United Kingdom). RNA was extracted with Trizol (Invitrogen, USA).

### RNA Analysis

Total RNA was extracted from tissue flash frozen in liquid nitrogen. cDNA was generated from 1 µg of RNA using Qiagen’s QuantiTect Reverse Transcription Kit. All splicing assays were done in at least three mice or more per group. mRNA splicing primers and conditions have been previous reported [2], [16], [18]–[20]. Gels were imaged and relative splicing levels were quantified using Image Quant5.1. Real-time qRT-PCR was done using the BioRad CFX Connect™ using manufacturer’s protocols and detected with SYBRGreen™ dye. Primer sequences and primer efficiencies can be found in Table S2 in File S1. *Gapdh* was use as our reference gene. The Livak (2^−ΔΔCT^) method was used to determine relative quantification. At least 3 mice per group were used for each analysis and each sample was done in duplicate to ensure accuracy.

### Electroporation of Skeletal Muscle

DM5^+/−^ mice were anesthetized by intraperitoneal injection of 100 mg/kg ketamine, and 1.25 mg/kg of midazolam. Tibialis anterior (TA) muscle was pretreated by intramuscular injection of bovine hyaluronidase (25 µl, 0.5 U/µl) (Sigma-Aldrich). Two hours later, 40 µg of EGFP-MBNL1 or EGFP plasmid in a total volume of 35 µl saline was injected using a 30-gauge needle. TA muscle was then electroporated using electrodes placed parallel to the long axis of the muscle. Electroporation parameters: Two sets of 5 square wave electrical pulses (switching polarity), 100 V, 20 ms pulses, 200 ms interpulse using the Electro Square Porator ECM 830 (BXT Harvard Apparatus) and the needle electrode. The investigators remained blinded to treatment status until EMG analyses were completed seven days later.

### Statistical Analysis

**Figure 1:**
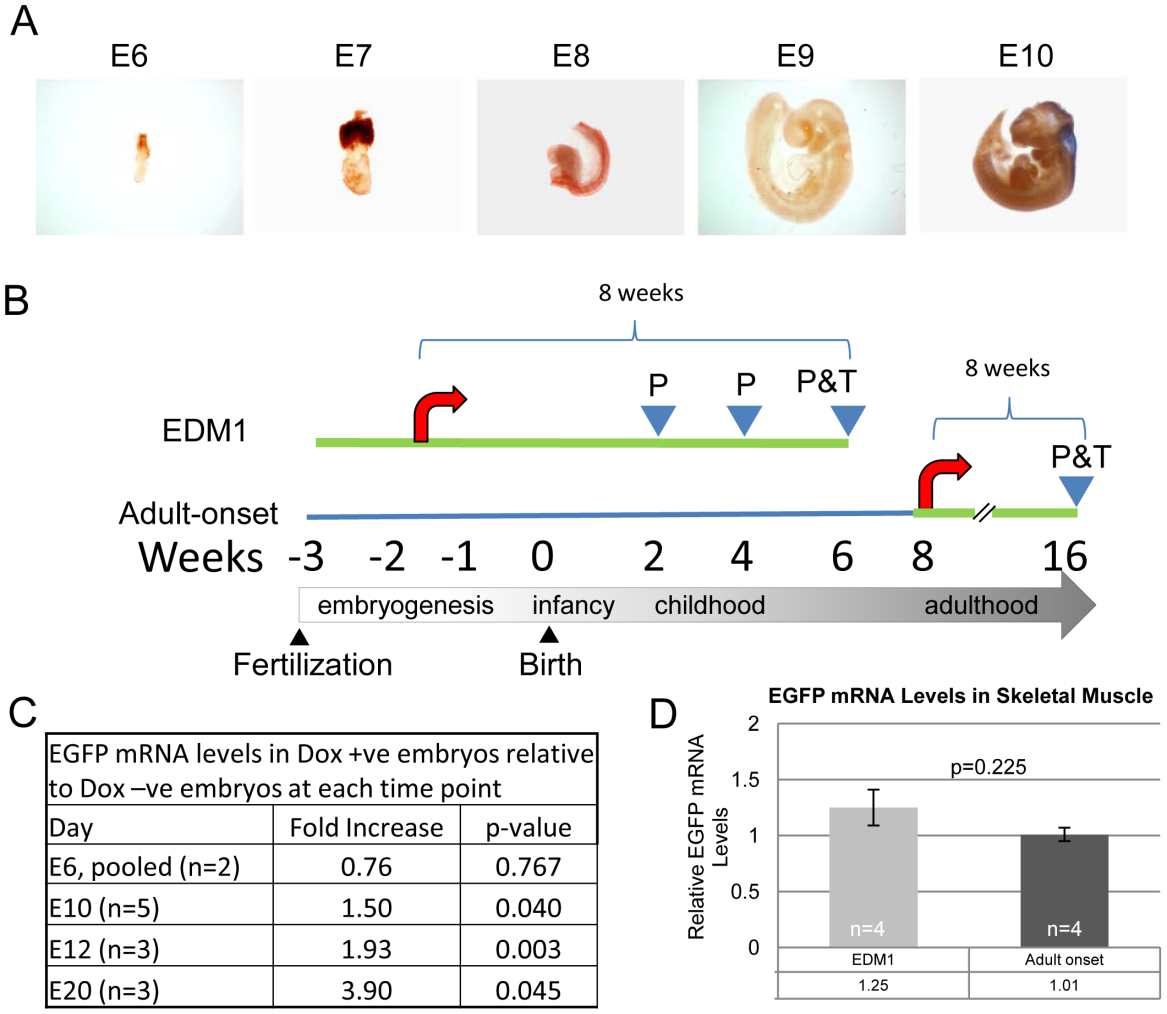
Generation of an early-induction DM1 mouse model. **A.** Whole mount *in-situ* hybridization of embryos at different time points showing onset of *Dmpk* expression (represented by the blue color) is visible only by the 10th day of development (E10). **B.** Schematic diagram representing the protocol used to generate and study the EDM1 mice and adult-onset mice. The green line represents administration of doxycycline. The red arrow represents induction of the transgene resulting in an increase in toxic RNA. Arrowheads with a P represent phenotypic analysis while the final arrowhead P&T represents phenotypic analysis and tissues collection. **C.** Induction of the transgene was measured in whole embryos by qRT-PCR at varying time points as indicated. Significant doxycycline mediated transgene induction starts at embryonic day 10, similar to results for endogenous *Dmpk* (see Figure 1A.). **D.** Graph of toxic RNA levels in skeletal muscle shows no significant difference between the EDM1 and adult-onset DM1 mice. Number of samples (n) provided on the graph, averages listed in the tables below the graphs, error bars are SEM. Two Sample T-Test was used to determine statistical significance.

**Figure 2:**
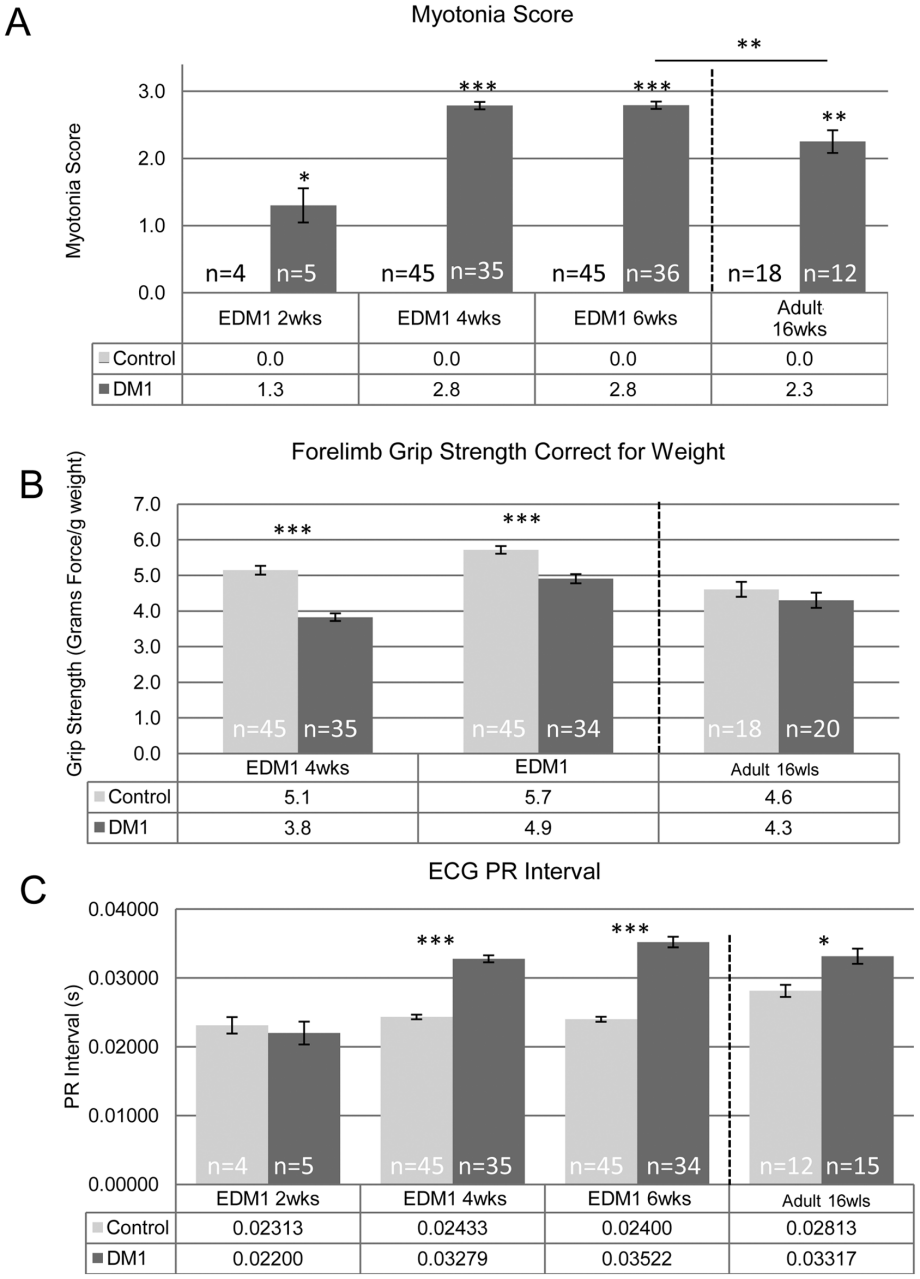
Characterization of EDM1 mice. **A.** Myotonia was measured and scored on a 3 point scale with 0 being no myotonia and 3 being strong, persistent, and prevalent myotonia. EDM1 mice show myotonia as early as 2 weeks of age and reached maximal severity by 4 weeks. **B.** EDM1 mice have a reduction in grip strength as early as 4 weeks of age while the adult-onset mice did not show a significant reduction. **C.** PR interval was measured in the EDM1 mice. No statistical difference was measured at 2 weeks of age; however the subsequent time points showed a significant lengthening of the interval. Similar but less severe results were observed in the adult-onset DM1 model. Number of samples (n) provided on the graph, averages listed in the tables below the graphs, error bars are SEM. Two Sample T-Test was used to determine statistical significance with p-values of 0.05 (*), 0.01 (**) or <0.001 (***) displayed.

**Figure 3:**
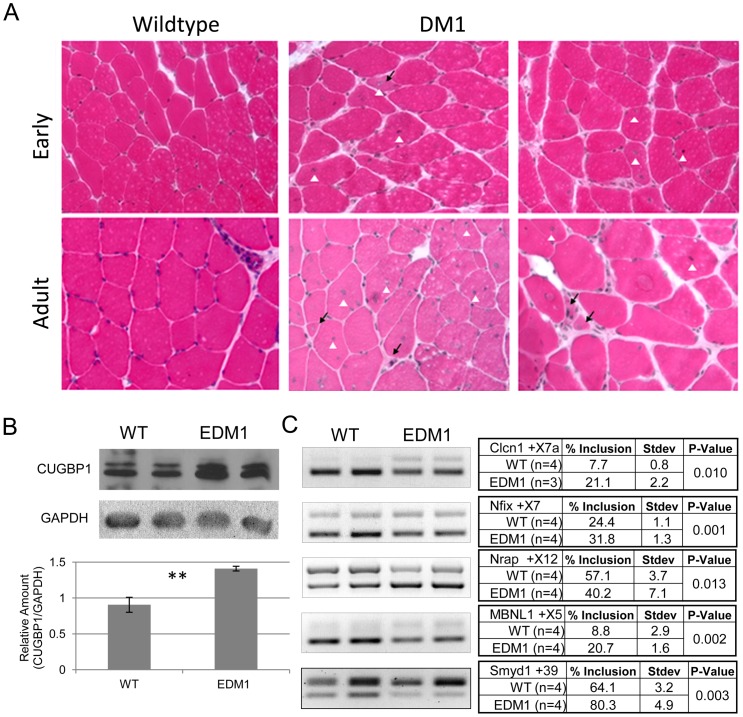
Histopathology, RNA splicing and CUGBP1 are all altered in the EDM1 mice. **A.** H&E staining shows uniform fibers and peripheral nuclei in the wildtype sections. With RNA toxicity, there is an increase in central nuclei (white arrowhead), increase in fiber size variability, and of atrophic fibers (black arrow). **B.** CUGBP1 is increased in the skeletal muscles of EDM1 mice. **C.** *Clcn1*, *Nfix*, *Nrap*, *Mbnl1*, and *SmyD1* splicing alterations found in human DM1 are present in the EDM1 mice. Number of samples (n) provided in the tables, averages listed in the tables along with standard deviation. Two Sample T-Test was used to determine statistical significance with p-values displayed.

**Figure 4:**
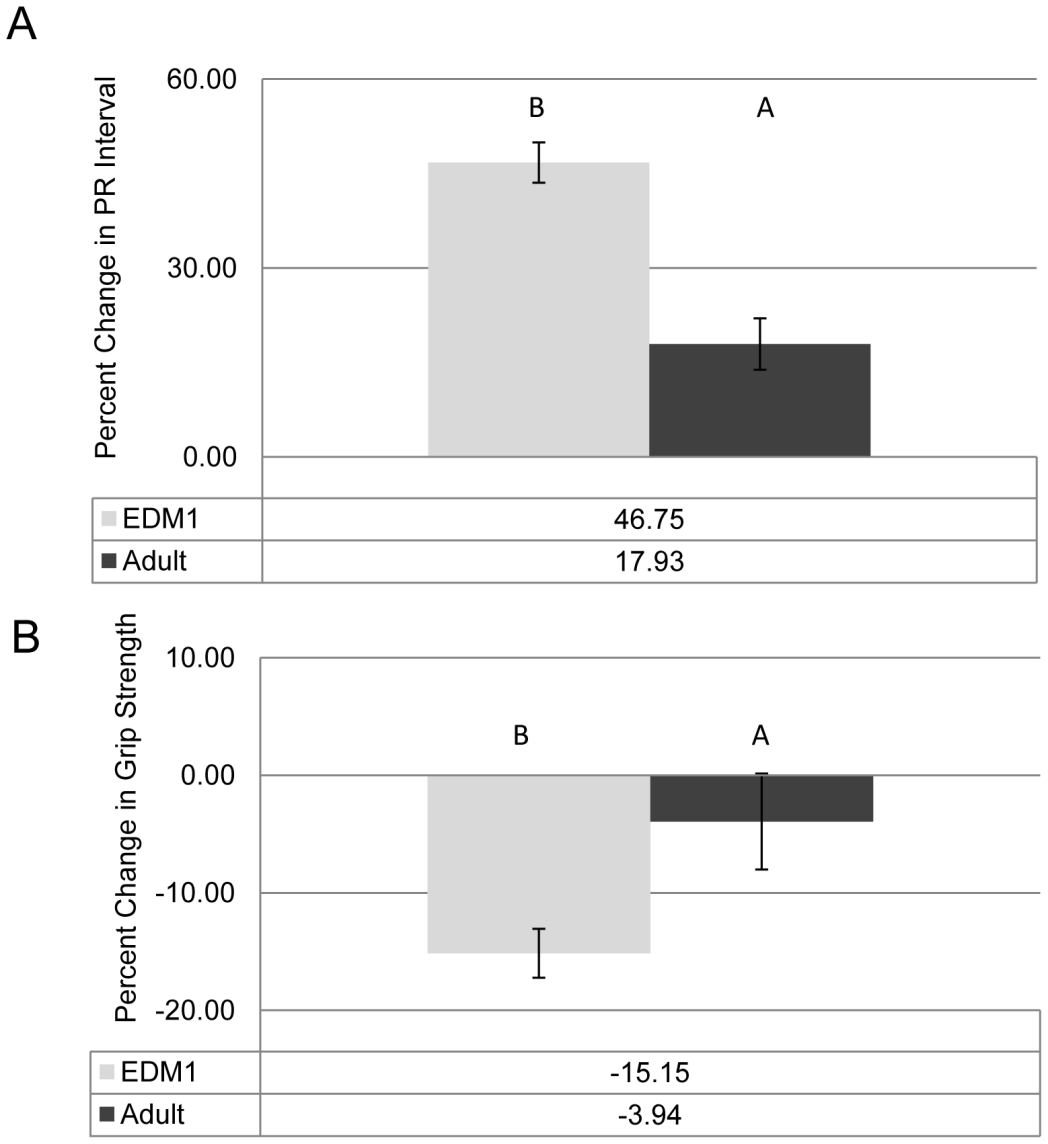
Age of onset of toxic RNA expression affects DM1 disease phenotypes. RNA toxicity animals were compared to control groups for both **A)** PR interval and **B)** grip strength. Effects on grip strength and PR interval were more severe in the early induction mice (EDM1) compared to the adult onset mice (Adult-onset DM1). Averages are listed in the tables below the graphs. Error bars are SEM. Groups that do not share a letter are significantly different (p<0.05) using a One-Way ANOVA with Tukey’s Multiple Comparisons. N = 10 or more for each control group used to generate the control average and N = 17 or more for each DM1 group.

Standard statistical methods were employed using the Minitab 16.1.0, produced by Minitab, Inc. Results are expressed as means plus or minus the standard error of the mean (SEM) or standard deviation (SD). Two-Sample T-tests, Mann-Whitney (for EMG Scores), and Wilcoxon Signed Rank Test (for EMG scores) were utilized in Figures 1 and 2. A one-way ANOVA with Tukey’s multiple comparison was used in Figure 3. Two-Sample T-tests, Mann-Whitney (for EMG Scores), and Wilcoxon Signed Rank Test (for EMG scores) were utilized in Figure 4. Statistical significance was set at a p-value of <0.05 (one asterisk), <0.01 (two asterisks) or <0.001 (three asterisks). Minitab statistical readouts can be found in Table S3 in File S1.

## Results

### Generation of an Early-onset Model of RNA Toxicity

As a prelude to generating an early-induction DM1 (EDM1) mouse model, we investigated the expression of the endogenous *Dmpk* gene and the behavior of the induced transgene during embryonic development. Using RNA *in-situ* hybridization we found that the endogenous *Dmpk* gene becoming active around embryonic day 10 (Figure 1A), as previously reported [21]. To assess the temporal expression of the inducible transgene and endogenous *Dmpk*, we bred FVB female mice with DM5^+/+^ males. The FVB females were either administered doxycycline or not. Subsequently, DM5^+/−^ embryos were collected from timed pregnancies (days 6, 10, 12 and 20 of gestation) and RNA was extracted and analyzed by qRT-PCR for transgene and *Dmpk* expression. qRT-PCR confirmed negligible expression of endogenous *Dmpk* at embryonic day 6, and a noticeable increase by day 10 (as also seen by the in *in-situ* hybridization results, Figure 1A). By post-natal day 1, *Dmpk* expression increased a further two to three fold. With regard to the transgene we found some minimal leakiness in the embryos collected from the uninduced dams, as previously reported for tissues from adult mice [16]. Interestingly, even in the uninduced state there was a slight increase (1.5 to 3 fold) between day 6 and day 10 of embryogenesis, paralleling the increase in endogenous *Dmpk*. In the induced state, the difference in transgene expression between day 6 and day 10 ranged between 4 to 8 fold. There was little if any induction of the transgene at day 6 with doxycycline administration, but significant induction by day 10 (Figure 1C), mirroring the behavior of endogenous *Dmpk*.

To generate EDM1 mice, wildtype (WT) and DM5 hemizygous animals were mated (for full details on the genotype and breeding strategy refer to the methods section). In the resulting litters half the mice carried the transgene and the other half were used as control littermates. Doxycycline was administered at the time of mating and maintained throughout gestation, birth, and rearing, up to 6 weeks after birth. Thus total transgene induction for these mice is approximately 8 weeks (∼12 days during gestation and 6 weeks after birth). To evaluate the severity of the disease phenotype we measured myotonia, grip strength, and PR interval in these mice every 2 weeks after the mice reached 2 weeks of age (Figure 1B). Likewise, adult-onset mice were induced starting at 2 months of age for a total of 8 weeks and phenotyped at the end of their 8 week induction (Figure 1B). In this way we ensured comparison of mice with similar duration of toxic RNA exposure. When toxic RNA levels were measured by qRT-PCR in the skeletal muscles of the EDM1 and adult-onset DM1 mice at the final time point, we found that they were equivalent in these two groups (Figure 1D).

Total body weight was measured at 8, 14, and 21, 30, and 44 days after birth. At no time were there any statistical differences in the weight of the EDM1 mice as compared to the WT control littermates. Weight was also measured during each measure of grip strength with averages listed in Table S1. Myotonia was measured using electromyography (EMG) and scored as previously reported [16]. In the EDM1 mice at two weeks of age only weak and intermittent myotonia was detected (median score of 1/3), however it increased in severity and intensity at successive time points (reaching a median of 3/3 by 4 weeks) (Figure 2A). Though grip strength increased in all groups with normal development, EDM1 mice had significantly weaker forelimb grip strength at both 4 and 6 weeks of age as compared to the control littermates (Figure 2B). Also, in the EDM1 mice, the PR interval (the time the electrical impulse takes to travel from the sinus node through the AV node) began to lengthen and was statistically increased by 4 and 6 weeks of age (Figure 2C). This lengthening is indicative of the development of cardiac conduction defects in these EDM1 mice.

At the end of six weeks, tissues were collected and muscle histopathology, splicing alterations, and protein changes were assessed. Muscle histology was compared between the EDM1 and control littermates. The EDM1 mice had an increase in centrally nucleated fibers, a common feature of myotonic dystrophy. Additionally there was a greater degree of fiber size variability and fiber atrophy as compared to the control littermates; similar features were observed in the adult-onset DM1 model (Figure 3A).

Sequestration of MBNL1 in RNA foci and the increase in CUGBP1 are cardinal features of the human DM1 disease pathology. The EDM1 mice, like previous results from our adult-onset DM5-313 mice, have increased CUGBP1 as compared to their control littermates (Figure 3B) [2], [16]. A number of splicing targets are known to be incorrectly spliced in DM1 due at least in part to the altered levels of MBNL1 and CUGBP1. Splicing targets known to be misregulated in patients with DM1 were examined in the EDM1 mice [2], [16], [18]–[20]. We found a robust and statistically significant change of 13.5% increase in exon 7a inclusion in *Clcn1*, a 7.4% increase in *Nfix* exon 7 inclusion, a 16.2% increase in *Smyd1* exon 39 inclusion, a 16.9% decrease in *Nrap* exon 12 inclusion, and a 11.9% increase in *Mbnl1* exon 5 inclusion (Figure 3C). Some of these targets such as *Mbnl1* exon 5, *Clcn1* exon 7 and *Nfix* exon 7 are known to be specifically regulated by MBNL1, suggesting that the EDM1 mice containing the DM5 transgene do have MBNL1 sequestration, even if it is not evident in discrete nuclear foci [18], [22], [23].

### Parental Contribution Plays No Role in the EDM1 Phenotype

While congenital DM1 is almost exclusively inherited from the maternal line, childhood-onset myotonic dystrophy can be inherited from either the maternal or paternal lines [9], [10], [13], [14]. To determine if the disease status of either parent affected the disease severity in the pups, wildtype females or males were mated with affected DM5 females or males (i.e. induced adult-onset DM1 mice). The resultant pups were either WT or EDM1, and were conceived, gestated and born from either a RNA toxicity-affected dam (i.e. an adult-onset DM1 mouse) or a healthy-normal dam (i.e. a wildtype mouse). As the DM5 transgene does not contract or expand in successive generations or in different tissues, the resulting pups all had the same genetic background, allowing for a straightforward comparison to determine parent of origin effects.

Forelimb grip strength, myotonia, and PR interval were compared between the EDM1 mice from the affected or unaffected dams at 4 weeks of age. We found that there was no difference in any measurement between the two groups (Table 1). Additionally there was no difference in the survival of pups born from affected or unaffected dams.

**Table 1:** Lack of parent of origin effect in the EDM1 mice.

	4 week old EDM1 Pups from		
	Normal Dam (n = 12)	Affected Dam (n = 23)	p-value
Grip Strength (g force/g weight)	3.6+/−0.5	3.9+/−0.7	0.15
PR Interval (s)	0.0325+/−0.0030	0.0329+/−0.0031	0.69
Myotonia (Score 0–3)	2.8+/−0.4	2.8+/−0.3	0.95

Mice born from a sick or healthy dam were not statistically different with regard to grip strength, PR interval or myotonia score at 4 weeks of age.

### Early Disease Induction Results in a More Severe Phenotype

To test if age of onset of RNA toxicity affected the phenotype observed in our mice, we compared the EDM1 mice that had been induced from conception to six weeks of age with hemizygous adult DM5 mice that were induced beginning at two months of age for a total duration of eight weeks. Both groups were exposed to RNA toxicity for an equivalent period of time (Figure 1B). A comparison of the forelimb grip strength, myotonia, and PR interval between control littermates and DM5 mice was undertaken. However, since grip strength and PR interval change with age, we compared percent change after normalizing the EDM1 and adult-onset DM1 mice to their normal littermates. The EDM1 mice had a statistically significant increase in disease severity as compared to the adult onset mice in PR interval, with an increase of 46.75% in EDM1 compared to 17.93% in adult-onset DM1 (Figure 4A). Additionally the myotonia score was slightly worse with an average score of 2.8/3 in EDM1 compared to 2.3/3 in adult-onset DM1 (Figure 2A). Loss of grip strength was also more severe (-15.15% in EDM1 compared to −3.94% in adult-onset DM1 mice) (Figure 4B). Interestingly skeletal muscle histology had a slightly milder muscle histopathology (Figure 3A) in the EDM1 mice as compared to the adult onset DM1 mice. This suggests that though the timing of toxic RNA expression during development can impact disease severity, not all aspects of the DM1 phenotype are necessarily worsened by this early exposure (e.g. muscle histopathology). Alternatively, ongoing muscle development in the young mouse could compensate for some of the histopathology typically associated with RNA toxicity.

### Over Expression of MBNL1 in Skeletal Muscle of EDM1 Mice Partially Corrects Myotonia and Some Splicing Defects

**Figure 5:**
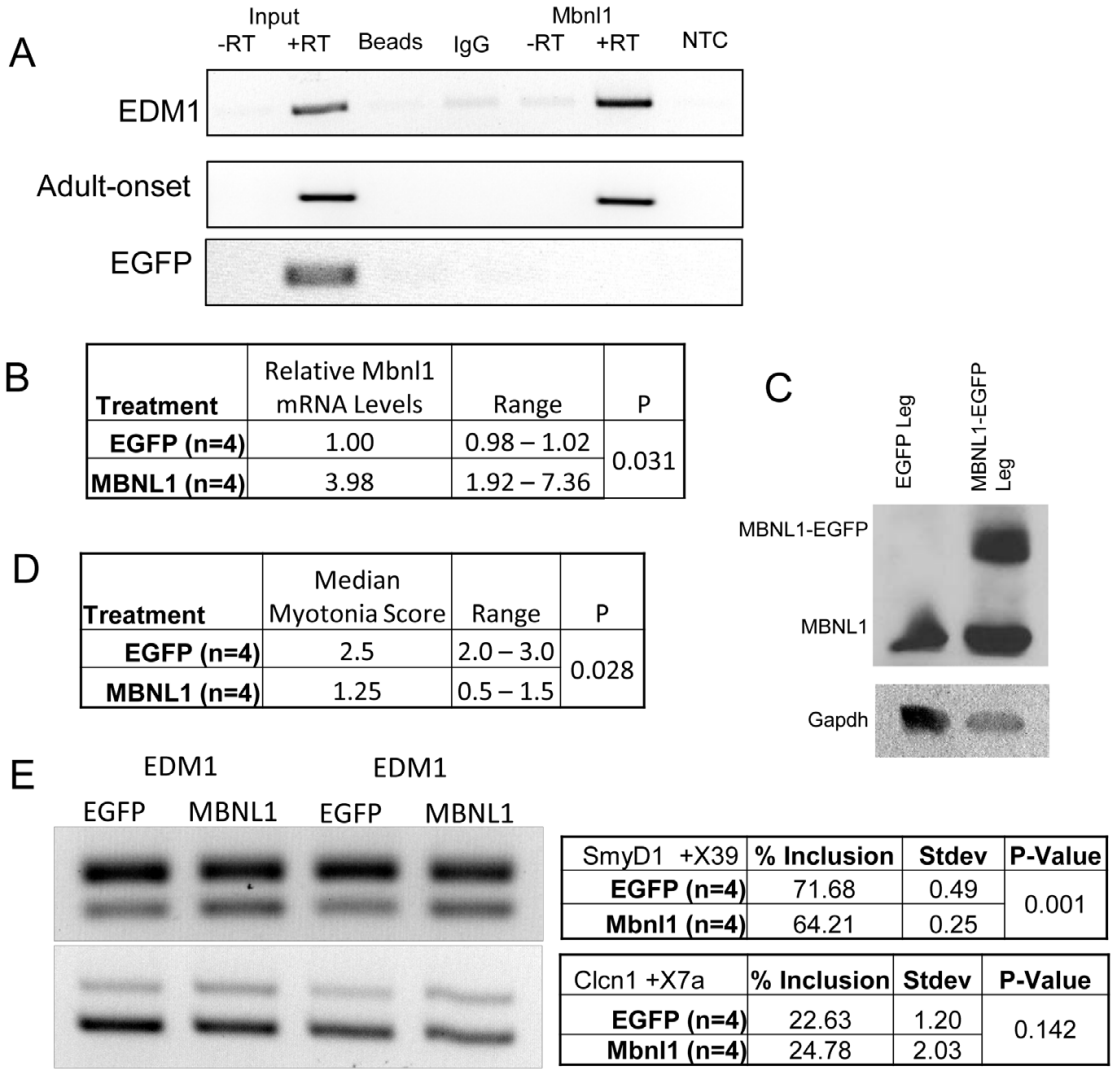
Partial Rescue of EDM1 phenotype in skeletal muscle by over-expression of MBNL1. **A.** RNA-IP using a monoclonal antibody against MBNL1 pulls down the GFP-*DMPK* 3′UTR mRNA in both the EDM1 and adult-onset DM1 mice but not mice expressing EGFP only. Beads, IgG and RT -ve controls were used to show MBNL1 specificity. EGFP was amplified to detect the transgene. **B.** MBNL1 was confirmed to be up-regulated by qRT-PCR and **C.** by western blot analysis in the MBNL1 treated leg but not the EGFP treated leg. **D.** Median myotonia score in EDM1 mice expressing MBNL1 and EGFP in their TA muscles shows a slight reduction in myotonia due the presence of MBNL1 but not EGFP. **E.** mRNA splicing was also partially improved for *SmyD1*. *Clcn1*-exon7a showed no change. Number of samples (n) provided along with average or median in the tables along with standard deviation. Two Sample T-Test was used to determine statistical significance with p-values displayed.

While the DM5 mouse model does not have visible MBNL1-RNA foci it is still possible that MBNL1 is being sequestered by the toxic RNA, which is known to be highly expressed in our model [2], [16]. To check for an *in vivo* interaction between the DM5 *DMPK* 3′UTR mRNA and MBNL1, we performed RNA immunoprecipitation (RNA-IP) on protein extracts from skeletal muscles expressing the DM5 transgene, using an MBNL1 specific antibody (Figure 5A). The transgene mRNA was specifically immunoprecipitated by the antibody, but not in the bead or IgG control, demonstrating a strong physical interaction between the MBNL1 protein and the transgene RNA (Figure 5A). Additionally, protein extracts from the skeletal muscles of a mouse over-expressing GFP without the *DMPK* UTR showed no interaction of the *GFP* mRNA with MBNL1. It is thus possible that while RNA foci are not visible, the toxic RNA could still be sequestering MBNL1 in the DM5 mice.

MBNL1 sequestration induced mRNA mis-splicing is thought to contribute significantly to the varied phenotypes associated with myotonic dystrophy. MBNL1 knockout mice show many of the same splicing problems and some of the phenotypic abnormalities associated with DM1, such as myotonia and cataracts. Additionally, over-expression of MBNL1 or liberation of MBNL1 from ribonuclear foci has been show to correct the altered splicing and myotonia in DM1 cellular and animal models [24], [25].

To determine if the EDM1 mice could be rescued by MBNL1, we over-expressed a MBNL1-EGFP fusion protein by electroporation gene transfer into the TA muscle of EDM1 mice. EGFP alone was electroporated into the contralateral TA muscle as a control. One week after electroporation, myotonia was assessed in both the control and MBNL1-EGFP expressing legs. We verified that MBNL1-EGFP mRNA and protein were being expressed in electroporated TA muscle by qRT-PCR and western blots analysis (Figure 5B and 5C). Levels likely fluctuated between mice, due to the limited area of expression and mosaic expression pattern that results from using this method. However, in all cases the MBNL1 transfected leg showed higher levels of MBNL1 then the control leg. Even though electroporation only provides a temporary and patchy expression of recombinant protein, a small but significant difference in myotonia between the control and MBNL1 treated muscle was detected in the EDM1 mice (Figure 5D). The average median score for myotonia was 2.5/3 for the TA expressing EGFP as compared to an average median score of 1.25/3 for the TA that expressed MBNL1-EGFP. Interestingly, when we looked at mRNA splicing, we did not see a reduction of Clcn1 exon 7a splicing, but this may likely be due to the relatively small treatment area as compared to the entire muscle from which the extracts were made. However we did see small but significantly change in another mRNA splicing target *SmyD1* exon 39 (Figure 5E). Full correction of the splicing defects and myotonia was likely not achieved due to the mosaic expression pattern resulting from electroporation [26], [27]. However, even the partial rescue of the skeletal muscle phenotypes observed in the EDM1 mice by MBNL1 over-expression highlights the important role proper MBNL1 expression plays in the DM1 phenotype.

## Discussion

The early-induction myotonic dystrophy model described in this study represents the first early-onset RNA toxicity model for DM1 with a multisystem phenotype that is measurably more severe than its adult onset counterpart. DM1 is considered to be one of the most clinically variable disorders known. There is still only a limited understanding of what molecular events lead to the many disease phenotypes, and even less is known about the basis of their variability. Though there is a general correlation between disease severity and the length of the CTG expansion found in leukocytes, it is by no means highly predictive. In individuals with DM1, repeat lengths vary from person to person [8]. Additionally, variations in the repeat lengths occur between the different tissues of an affected individual and can even change over time [28]. Though this variation may explain much of the phenotypic variability observed in myotonic dystrophy, it is still unclear exactly why increased (CUG) repeats result in a disease where age of onset, and ultimately phenotypic severity, can vary so greatly. One hypothesis is that a threshold of (CUG) RNA toxicity has to be reached before cellular coping mechanisms are overwhelmed, subsequently resulting in overt disease manifestations. This could be attained by a variety of mechanisms, but one possibility is that the affected individual is conceived from a germ cell with a very large CTG expansion, as is often seen in congenital DM1. As D*MPK* is clearly expressed from early stages of embryogenesis (Figure 1A), this implies that individuals with very large expansions would reach the threshold earlier. That is, RNA toxicity would start earlier. Alternatively, in individuals with smaller CTG expansions, this threshold may not be reached until adulthood, when repeated cycles of expansion due to repeat instability have pushed the CTG expansion beyond the threshold for RNA toxicity in affected tissues. This could have very distinct consequences as the onset of RNA toxicity at different stages of development may result in very different molecular and phenotypic outcomes.

With our mouse model, there is no variability in the number of repeats in various tissues, or with time. Unlike in human patients, the (CTG)_n_ tract does not change over multiple generations; and the toxic stress from the RNA occurs only after activation of the transgene by administration of doxycycline. However, unlike other mouse models of RNA toxicity, in which the role of age of onset of RNA toxicity cannot be directly evaluated, we have the distinct advantage of inducing RNA toxicity at will. This allowed a direct examination of the effect of RNA toxicity at different points in development while avoiding the impact of complicating variables, such as repeat length variability and somatic instability, both of which occur in the human situation and in some mouse models. Does crossing the threshold of RNA toxicity earlier in development result in a more severe disease purely due to the timing of disease onset? In this study we have begun to answer this question using the EDM1 and adult-onset DM1 mice which have an unchanging repeat length and are not subject to genetic anticipation between successive generations.

The results from our experiments provide evidence that the age of onset of pathologically significant levels of RNA toxicity can affect disease severity. The EDM1 mice, in which the toxic RNA was expressed during early growth and development, disease readouts like myotonia, PR interval, and to a lesser extent, muscle strength were affected to a greater extent than in mice that were initially exposed to RNA toxicity as adults (Figure 2 and Figure 4). Interestingly, not all disease symptoms readouts were as affected. For instance, muscle histology in the EDM1 mice was generally milder than the mice with adult onset disease (Figure 3A). This could be due the fact that the younger, EDM1 mice were still growing and developing and were able to compensate for some of the disease pathology. This is reminiscent of the situation where children with severe DM1 often get better before developing the progressive deterioration associated with adult-onset DM1 [9], [10]. Perhaps the complex sets of molecular pathways that are altered in DM1 are different when the toxic RNA is present in the developing individual as compared to the adult.

The ultimate goal for generating a mouse model of disease is to develop a tool to further understand the disease in all its many forms, to uncover molecular mechanisms and use that knowledge to develop and test therapies. For instance, by taking advantage of the inducible nature of the transgene, in this study we were able to clearly demonstrate for the first time, the effects of age of onset of RNA toxicity on disease outcome. Though our model is unusual in that it expresses a *DMPK* 3′UTR with only (CTG)_5_, it exhibits the multisystemic features of the disease as well as many of its molecular hallmarks. This is consistent with the preceding hypothesis of (CUG) RNA toxicity thresholds, which in our DM5 model may be achieved by high over-expression of a *DMPK* 3′UTR (CUG)_5_ mRNA. One of the drawbacks of this model has been the lack of RNA foci, but now with the result from the RNA-IP showing an interaction of MBNL1 with the DM5-*DMPK*-3′UTR mRNA (Figure 5), this suggests that MBNL1 sequestration may occur in the DM5 mouse model and that this binding leads to phenotypic consequences. This notion is further supported by the results using the EDM1 mouse model where we were able to show that over-expression of MBNL1 was capable of partially correcting myotonia and some mRNA splicing defects (Figure 5), akin to the results obtained using the HSA-LR mice [24]. This makes the adult-onset DM1 and EDM1 mouse models, which have robust multi-systemic phenotypes and utilize the doxycycline inducible EGFP *DMPK* 3′UTR, a useful model to test many of the ever increasing number of therapeutic options aimed at treating myotonic dystrophy.

## Supporting Information

File S1 **Supporting Information for materials and methods.** Table S1, Mass (grams) of the different groups of mice. Table S2, Sequences of PCR primers and Efficiencies of Relevant Real Time PCR reactions. Table S3, Details of Statistical Analyses.
(DOCX)
