# Supplementary material for: Age of Onset of RNA Toxicity Influences Phenotypic Severity: Evidence from an Inducible Mouse Model of Myotonic Dystrophy (DM1)
Source: PLoS One. 2013 Sep 5;8(9):e72907. doi: 10.1371/journal.pone.0072907 (PMC3764231; doi:10.1371/journal.pone.0072907)
Supplement: File S1 — Supporting Information for materials and methods. Table S1, Mass (grams) of the different groups of mice. Table S2, Sequences of PCR primers and Efficiencies of Relevant Real Time PCR reactions. Table S3, Details of Statistical Analyses. (DOCX) [file pone.0072907.s001.docx]

**Table S1. Mass (grams) of the different groups of mice.**

| **Age of mice** | **Induced Control Littermates** | | **Induced**  **DM5^+/-^ Mice** | |  |
| --- | --- | --- | --- | --- | --- |
|  | Average | SD | Average | SD | p-value |
| 4wks | 16.4 | 2.4 | 16.3 | 2.6 | 0.86 |
| 6wks | 20.4 | 2.3 | 21.4 | 2.2 | 0.07 |
| 16wks | 30.9 | 4.5 | 28.5 | 3.0 | 0.23 |

**Table S2. Sequences of PCR primers and Efficiencies of Relevant Real Time PCR reactions.**

**Primers:**

*Mbnl1* F: CCTTCGAAAAGCTGCCAAGTT

*Mbnl1* R: CATGGCCATGTTCTTCTGCTG

*EGFP* F: GGGCACAAGCTGGAGTACAACTAC

*EGFP* R: ACTTGTACAGCTCGTCCATGC

*Dmpk* F: GACGTGGATGGGCAAACTGC

*Dmpk* R: GACAATAAATACCGAGGAATGTC

*Gapdh* F: AGGTCGGTGTGAACGGATTTG

*Gapdh* R: TGTAGACCATGTAGTTGAGGTCA

**Efficiencies of Real Time PCR reactions for various targets:**

*Mbnl1*: 100%

*EGFP*: 95%

*Dmpk*: 95%

*Gapdh*: 93%

**Table S3. Details of Statistical Analyses.**

*Statistical Analysis.* Standard statistical methods were employed using the Minitab 16.1.0, produced by Minitab, Inc.. Minitab statistical readouts can be found below:

**Two-Sample T-Test and CI: Grip Strength (Figure 2)**

Two-sample T for 4WT vs 4EDM1

N Mean StDev SE Mean

4WT 45 5.146 0.829 0.12

4EDM1 35 3.828 0.635 0.11

Difference = mu (4WT) - mu (4EDM1)

Estimate for difference: 1.319

95% CI for difference: (0.993, 1.644)

T-Test of difference = 0 (vs not =): T-Value = 8.06 P-Value = 0.000 DF = 77

Two-sample T for 6WT vs 6DM1

N Mean StDev SE Mean

6WT 46 5.714 0.727 0.11

6EDM1 34 4.906 0.750 0.13

Difference = mu (6WT) - mu (6DM1)

Estimate for difference: 0.808

95% CI for difference: (0.474, 1.142)

T-Test of difference = 0 (vs not =): T-Value = 4.83 P-Value = 0.000 DF = 69

Two-sample T for 16WT vs 16DM1

N Mean StDev SE Mean

16WT 18 4.608 0.892 0.21

16DM1 20 4.302 0.954 0.21

Difference = mu (16WT) - mu (16DM1)

Estimate for difference: 0.306

95% CI for difference: (-0.302, 0.914)

T-Test of difference = 0 (vs not =): T-Value = 1.02 P-Value = 0.314 DF = 35

**Wilcoxon Signed Rank Test: EMG Score (Figure 3)**

Test of median = 0.000000 versus median not = 0.000000

N for Wilcoxon Estimated

N Test Statistic P Median

2EDM1 6 6 21.0 0.036 1.500

4EDM1 35 35 630.0 0.000 2.750

6EDM1 36 36 666.0 0.000 2.750

16DM1 12 12 78.0 0.003 2.250

Mann-Whitney Test and CI: EMG Score EDM1 vs ADM1

N Median

6EDM1 36 3.0000

16DM1 12 2.2500

Point estimate for ETA1-ETA2 is 0.5000

95.1 Percent CI for ETA1-ETA2 is (-0.0001,0.9998)

W = 999.0

Test of ETA1 = ETA2 vs ETA1 not = ETA2 is significant at 0.0055

The test is significant at 0.0020 (adjusted for ties)

**One-way ANOVA: EDM1, JVDM1, ADM1 Grip Strength (Figure 4)**

Source DF SS MS F P

Factor 2 1794 897 3.89 0.025

Error 72 16590 230

Total 74 18384

S = 15.18 R-Sq = 9.76% R-Sq(adj) = 7.25%

Individual 95% CIs For Mean Based on

Pooled StDev

Level N Mean StDev ----+---------+---------+---------+-----

EDM1 33 -15.15 11.94 (--------*--------)

JVDM1 17 -11.11 11.34 (-----------*------------)

ADM1 25 -3.94 20.38 (---------*----------)

----+---------+---------+---------+-----

-18.0 -12.0 -6.0 0.0

Pooled StDev = 15.18

Grouping Information Using Tukey Method

N Mean Grouping

ADM1 25 -3.94 A

JVDM1 17 -11.11 A B

EDM1 33 -15.15 B

Means that do not share a letter are significantly different.

Tukey 95% Simultaneous Confidence Intervals

All Pairwise Comparisons

Individual confidence level = 98.05%

EDM1 subtracted from:

Lower Center Upper ---------+---------+---------+---------+

JVDM1 -6.79 4.04 14.87 (----------*----------)

ADM1 1.58 11.20 20.82 (--------*---------)

---------+---------+---------+---------+

-10 0 10 20

JVDM1 subtracted from:

Lower Center Upper ---------+---------+---------+---------+

ADM1 -4.24 7.16 18.57 (----------*-----------)

---------+---------+---------+---------+

-10 0 10 20

**One-way ANOVA: EDM1, JVDM1, ADM1 PR Interval (Figure 4)**

Source DF SS MS F P

Factor 2 13374 6687 16.73 0.000

Error 62 24789 400

Total 64 38163

S = 20.00 R-Sq = 35.04% R-Sq(adj) = 32.95%

Individual 95% CIs For Mean Based on

Pooled StDev

Level N Mean StDev -----+---------+---------+---------+----

EDM1 34 46.75 18.73 (---*----)

JVDM1 16 57.72 25.78 (-----*-----)

ADM1 15 17.93 15.20 (-----*------)

-----+---------+---------+---------+----

16 32 48 64

Pooled StDev = 20.00

Grouping Information Using Tukey Method

N Mean Grouping

JVDM1 16 57.72 A

EDM1 34 46.75 A

ADM1 15 17.93 B

Means that do not share a letter are significantly different.

Tukey 95% Simultaneous Confidence Intervals

All Pairwise Comparisons

Individual confidence level = 98.08%

EDM1 subtracted from:

Lower Center Upper ---------+---------+---------+---------+

JVDM1 -3.61 10.97 25.54 (----*----)

ADM1 -43.73 -28.83 -13.93 (----*----)

---------+---------+---------+---------+

-30 0 30 60

JVDM1 subtracted from:

Lower Center Upper ---------+---------+---------+---------+

ADM1 -57.07 -39.79 -22.52 (-----*----)

---------+---------+---------+---------+

-30 0 30 60

**Mann-Whitney Test and CI: Myotonia Score (Figure 5)**

N Median

EGFP 4 2.500

MBNL1 4 1.250

Point estimate for ETA1-ETA2 is 1.500

97.0 Percent CI for ETA1-ETA2 is (0.500,2.500)

W = 26.0

Test of ETA1 = ETA2 vs ETA1 not = ETA2 is significant at 0.0304

The test is significant at 0.0275 (adjusted for ties)
